# Synthesis of new metastable nanoalloys of immiscible metals with a pulse laser technique

**DOI:** 10.1038/srep09849

**Published:** 2015-05-08

**Authors:** Zaneta Swiatkowska-Warkocka, Alexander Pyatenko, Franciszek Krok, Benedykt R. Jany, Marta Marszalek

**Affiliations:** 1 Institute of Nuclear Physics Polish Academy of Sciences, PL-31342 Krakow, Poland; 2 National Institute of Advanced Industrial Science and Technology, Tsukuba, 305-8565, Japan; 3 Marian Smoluchowski Institute of Physics Jagiellonian University, Krakow, 30-348, Poland

## Abstract

The generation of nanoalloys of immiscible metals is still a challenge using conventional methods. However, because these materials are currently attracting much attention, alternative methods are needed. In this article, we demonstrate a simple but powerful strategy for the generation of a new metastable alloy of immiscible metals. Au_1−x_Ni_x_ 3D structures with 56 at% of nickel in gold were successfully manufactured by the pulsed laser irradiation of colloidal nanoparticles. This technology can be used for preparing different metastable alloys of immiscible metals. We hypothesise that this technique leads to the formation of alloy particles through the agglomerations of nanoparticles, very fast heating, and fast cooling/solidification. Thus, we expect that our approach will be applicable to a wide range of inorganic solids, yielding even new metastable solids that fail to be stable in the bulk systems, and therefore do not exist in Nature.

The synthesis of bimetallic particles with well-defined architectures (e.g. core-shell or multishell structures, hollow structures, heterostructures, alloys) has attracted considerable attention because they show multiple functionalities and outstanding catalytic, magnetic and photonic properties^1^,^2^,^3^. Among these architectures, alloy particles are of particular interest because of their excellent chemical, optical, catalytic, magnetic, mechanical and tribological properties, and widespread use in numerous applications^4^,^5^,^6^,^7^,^8^,^9^,^10^,^11^,^12^. So far, most of the published reports have concentrated on the synthesis of alloy particles made from bulk miscible metals (e.g., PtPd, CuPd)^10^,^11^, but the studies on alloy particles with bulk immiscible metals (e.g., AuNi) are limited^4^. Accessing composites within the miscibility gaps is important because such phases offer interesting synergistic properties that cannot be achieved in phase segregated mixtures^12^.

Bulk Au and Ni have a large immiscibility gap according to its phase diagram^13^. The room-temperature solubilities of both Au in Ni and Ni in Au are very small, of the order of 0.01 at%. Since they also have significantly different reduction potentials, chemical synthesis is difficult to perform. AuNi solid solutions have been prepared using an ion beam mixing technique or an electrodeposition technique^14^,^15^, but the obtained alloys are formed as films or on the substrates. Until now, only a few studies reported synthesis of AuNi alloy nanoparticles by chemical reduction methods^16^,^17^. The synthesis of stable, homogeneous AuNi alloy particles formed as isolable solids has been so far limited.

The laser ablation of a bulk metal or an alloy target in a liquid has been shown to be an effective approach for synthesis of nanoalloys (e.g., AuAg^18^, AuFe^19^, PtIr^20^, NiFe, and SmCo^21^ nanoparticles, AgNi nanorods^22^, or AgCu nanowires^23^). Jakobi *et al.*^20^,^21^ demonstrated that chemical composition of alloy nanoparticles produced by the laser ablation depends on the composition of a target, and the stoichiometry of alloy nanoparticles and an alloy target is maintained only for metals with similar enthalpy of vaporization. Recently, Zhang *et al.*^24^, and Malvija and Chattopadhyay^25^ showed that composition of PtAu and AgCu alloy nanoparticles can be tuned by varying the ratio of metals in the targets. Amendola *et al.*^26^ demonstrated that elemental composition of AuFe nanoalloys can be adjusted by varying the proportion of ethanol and distilled water in the liquid solution. However, the laser ablation products demonstrate relatively wide size distributions and low yields, which is not favourable for some applications. In addition, preparation of the homogeneous alloy targets, especially from bulk immiscible metals, is very challenging. In this context, the pulsed laser irradiation of colloidal nanoparticles in solution^27^ seems to be a good alternative. Nanoparticles of AuCo have been obtained by the laser irradiation of Au and Co-oxide dispersed in ethanol solid solutions^28^. Prolongated irradiation time increases the relative amount of the Co phase up to a maximum value of 31 at% in the Au_1−x_Co_x_ particles. In this article, the same scheme was applied to the Au-Ni system. A nanosecond Nd:YAG laser was used to irradiate the mixture of colloidal solutions of gold and nickel oxide, resulting in Au_1−x_Ni_x_ 3D formation.

## Results and Discussion

**Figure 1:**
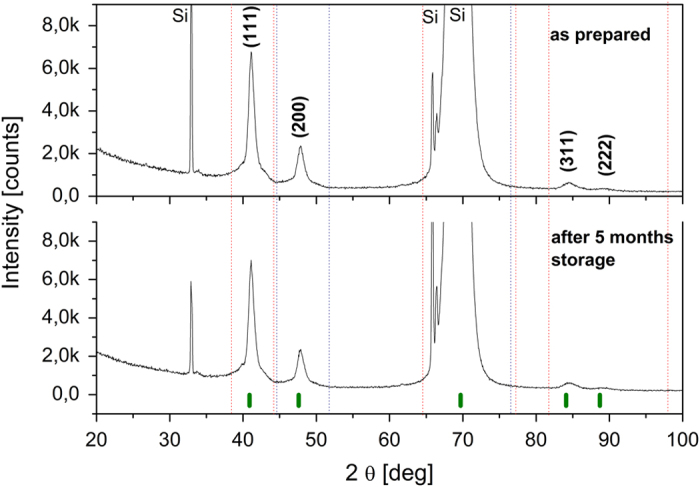
XRD patterns of Au_1−x_Ni_x_ particles (top) after the pulsed laser irradiation (195 mJ/pulse cm^2^, 1 h, solvent: ethanol, molar ratio of Au:NiO 1:1), and (bottom) after 5 months storage at room temperature. The vertical lines at the bottom indicate the diffraction angles of allowed Bragg reflections of the AuNi alloy (PDF: 01-072-9108)^30^, (the peak positions from Au (JCPDS 4-0784) and Ni (JCPDS 4-0850) are marked by dashed vertical lines in red, and blue, respectively).

The XRD pattern of the as-synthesized AuNi particles is shown in Fig. 1. No trace of signal coming from pure elements was observed for the sample. Besides the peaks coming from (211) and (422) planes of the Si(100) substrate we observed only two diffraction peaks at 41.16° and 47.88°. Their position was completely different from the position of the strongest peaks which could originate from the pure Au and Ni metals. Based on Vegard’s law, the diffraction peak of the alloy should be positioned between the corresponding peaks of metallic components. Since the peak at 41.16^o^ is situated between (111) peaks of Au and Ni (38.18° and 44.51°, respectively, data taken from JCPDS 4-0784 and 4-0850), we attributed it as the (111) peak of the AuNi alloy. The same strategy was applied for the peak at 47.88°, which was assigned to the (200) planes of the AuNi alloy. The results show that the sample conserves the fcc structure of the components with space group Fm-3m and the lattice constant a, calculated from the peak analysis to be 3.810 ± 0.004 Å. The contraction of the lattice constant in comparison to that of pure Au (a = 4.070 ± 0.005 Å) indicates diffusion of Au and Ni, and formation of the solid solution Au_1−x_Ni_x_. Lattice parameters were calculated using Rietveld refinement^29^ with the Fullprof program. Using the lattice parameter obtained for Au_1−x_Ni_x_, and the dependence of the lattice constant on the concentration of Ni in AuNi presented by Bienzel *et al.*^30^, we estimated the concentration of nickel in the particles as 56 ± 1 at%. The XRD measurements of the samples were repeated 5 months after the synthesis and confirmed the perfect stability of the Au_1−x_Ni_x_ alloy.

**Figure 2:**
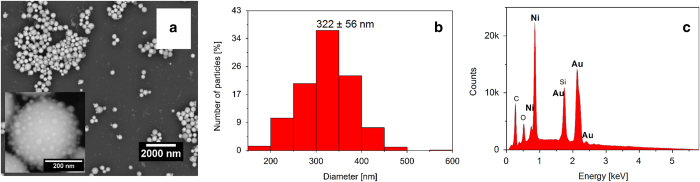
SEM, histogram, and EDS results. (**a**) SEM image of as-synthesized AuNi particles. Inset: magnified BSE image, (**b**) The particle size distribution based on SEM images. (**c**) SEM EDS analysis result.

Fig. 2a illustrates scanning electron microscopy (SEM) micrograph of as-prepared particles with an average diameter of 322 ± 56 nm (Fig. 2b). The back-scattered electrons (BSE) magnified image in the inset, which shows atomic number contrast, demonstrates that the composition is the same in all directions in the particle. The atomic ratio of Au:Ni determined by SEM EDS measurements (Fig. 2c) for the particles is 45:55, which is consistent with the results from XRD.

**Figure 3:**
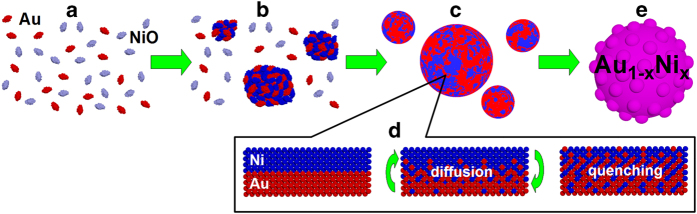
Illustration of Au_1−x_Ni_x_ nanoalloy formation by the pulsed laser irradiation of a mixture of Au and NiO colloidal nanoparticles.

It is well known that AuNi alloys are difficult to obtain, because Au and Ni are almost completely immiscible in the bulk phase. Here, however, we obtained Au_0.44_Ni_0.56_ alloy particles, rather than the more stable Ni@Au core-shell structures^31^,^32^. Why can AuNi alloy particles be formed using our technique? We hypothesise that the agglomerations of nanoparticles, very fast heating, and fast cooling are the main reasons (Fig. 3). Because both types of raw particles are small (Fig. 3a), they agglomerate very strongly, causing the distances between particles to become short (Fig. 3b). The raw nanoparticles adhere to each other, and the energy can be easily transferred from Au to NiO. NiO decomposes thermally to Ni by ethanol pyrolysis products^33^ (Fig. 3b). In the experiments conducted with a Nd:YAG laser, the agglomerates can be heated to a very high temperature, due to absorption of the laser-beam energy (Fig. 3c). The main difference between the pulsed laser irradiation and other synthetic methods is that in the pulsed laser irradiation the Au/Ni mixture can be heated to the temperature higher than the melting point, whereas under the normal experimental conditions the temperature is much lower and the particles can be heated only to the melting point. The pulsed heating (within 10 ns) leads to the very fast heating and to the high kinetic energy of the atoms. This energy can overcome the repulsion force between Au and Ni atoms^34^ which must be the reason for their immiscibility. As a result, mutual diffusion of Au and Ni takes place. The intermixed atoms may remain in the intermixed state, leading to the formation of a metastable phase. In our experiment very fast cooling/solidification process occurs, which takes 10^−5^-10^−6^ s^35^. Due to such a rapid quenching the Ni atoms cannot be ejected from the Au crystal lattice during the crystallization process. The situation may well be compared to a quenching process, in the sense that atoms are “frozen in” rapidly into a metastable state before equilibrium condition (such as a phase separation) is reached (Fig. 3d). AuNi alloy particles can be formed as a result of these processes (Fig. 3e).

**Figure 4:**
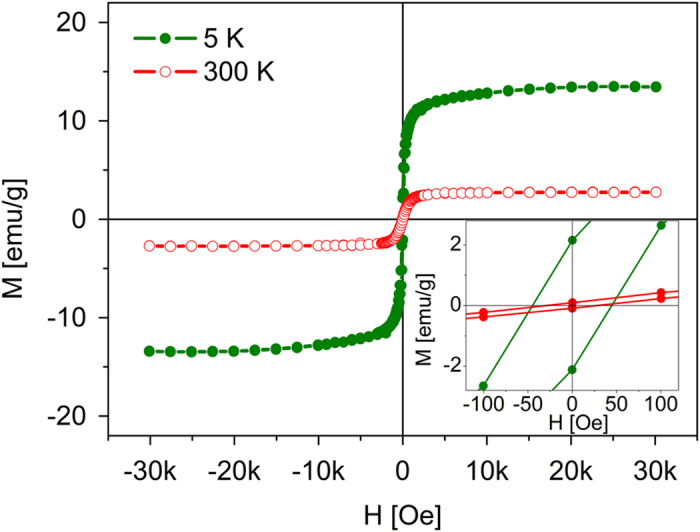
Magnetic hysteresis loops at 300 K and 5 K for Au_1−x_Ni_x_ particles. Inset shows enlargement of the loops for the zero value of the magnetic field.

To analyse the magnetic properties of the particles, we measured the magnetic hysteresis loops at 5 and 300 K with a superconducting quantum interference device (SQUID) (Fig. 4). The material exhibits a soft ferromagnetic state with small remnant magnetization (0.1 emu/g) and small coercivity (34 Oe) at room temperature, which is desirable for many practical applications, in which strong magnetic signals at small applied magnetic fields are advantageous.

**Figure 5:**
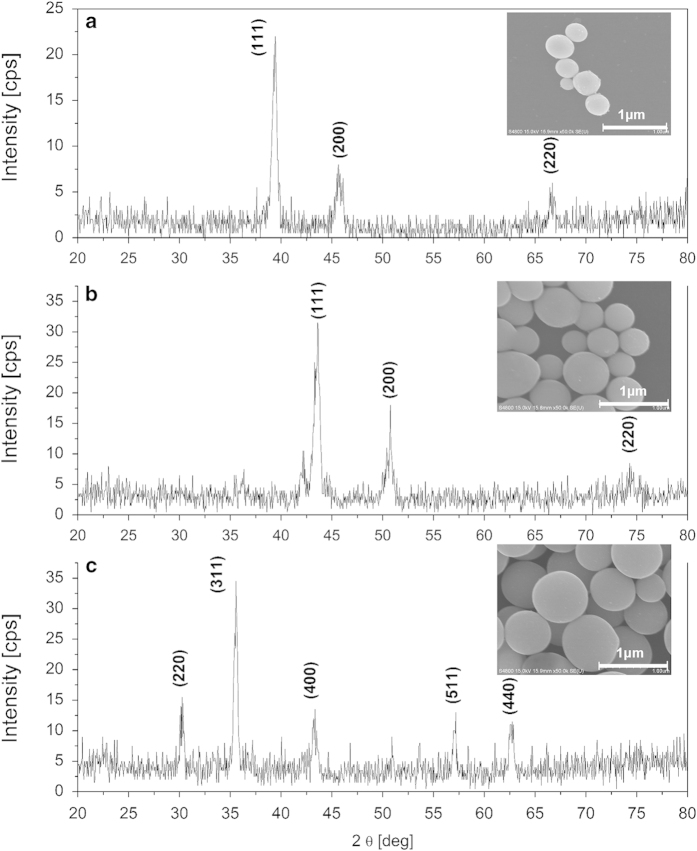
XRD patterns and SEM images of (a) Au_x_Co_y_ alloy^28^, (b) FeNi alloy (ICDD 00-0003-1016), (c) CuFe_2_O_4_ particles (ICDD 00-003-0870).

It should be noted that the AuNi binary system is a typical phase-separation system in a bulk phase diagram and the synthesis of stable, homogenous Au_1−x_Ni_x_ submicrometer spheres with 56 at% of nickel in gold via the laser irradiation is a significant result, unusual for other known techniques. Most importantly, the pulsed laser irradiation is suitable for preparing not only alloy particles made from the bulk miscible metals (e.g. FeNi) but also for the alloys that are immiscible under equilibrium (e.g., Au_x_Co_y_, Au_x_Fe_y_, x > y)^28^,^36^. Moreover, this technology is suitable for many others types of the composite particles of oxides, or semiconductors^27^,^37^,^38^,^39^. Figure 5 shows some typical examples of these materials.

In summary, by using Au-NiO as an example, we developed a new approach for the synthesis of Au_1−x_Ni_x_ alloy with the pulsed laser irradiation of nanoparticles colloidal solutions, which is substantially different from the conventional synthetic methods. Overheating of particles and a very rapid quenching process allow to overcome the repulsion force responsible for the immiscibility of Au and Ni. This technology could be used to prepare different metastable alloys of immiscible metals. Thus, we expect that our approach to the synthesis of alloy particles will be applicable to a wide range of inorganic solids, even yielding new metastable solids that are not stable in the bulk systems, and therefore do not exist in Nature.

## Methods

### Materials

The raw nanoparticles of gold (nanoparticles aqueous dispersion, 15 nm, 1000 ppm) were purchased from US-nano, and NiO nanoparticles (powder form, average size 20 nm, purity 99.5%) were purchased from Sigma Aldrich.

### Synthesis

The synthesis method of Au_1−x_Ni_x_ particles was described previously in reference 28Raw nanoparticles of gold (0.5 mM) and NiO (0.5 mM) were dispersed in ethanol (15 ml).

### Characterisation

The morphology and composition of the obtained gold/nickel particles were observed by a field emission scanning electron microscope (SEM; FEI Quanta 3D FEG) equipped with the EDAX EDS detector system. The average particle size was determined by measuring the diameters of 500 particles in the HR SEM image.

X-ray diffraction experiments in this study were performed with a two-circle laboratory diffractometer (Panalytical X'Pert Pro) using a standard θ-2θ geometry. For XRD measurements the solution with synthesized particles was dried on a Si (100) substrate. XRD patterns were taken using the Cu K_α_ line at 1.54 Å. Cu radiation obtained at 40 kV and 30 mA was converted into a parallel beam by incident beam optics with 0.5^ο^ divergence slit, parabolic graded W/Si mirror with an equatorial divergence less than 0.05^o^, and 0.04 rad Soller slit collimator. The axial width of the incident beam was restricted by the incident beam mask to 5 mm. The diffracted beam path was equipped with an anti-scatter slit and 0.04 rad Soller slit collimator. The signal was collected by solid state stripe detector with a graphite monochromator. The smallest step size of 2θ that can be defined with this optics was 0.0021^o^ for a goniometer radius of 240 mm. NIST LaB_6_ line profile standard SRM660a was measured to determine the angular resolution of the instrument. Based on these measurements, the instrumental peak broadening of 0.05^o^ was obtained for the 2θ range from 20^o^ to 100^o^ and all diffraction patterns were collected with a step size of 0.05^o^. Fullprof software (free software from https://www.ill.eu/sites/fullprof/php/tutorials.html) was used for peak fitting and to evaluate the lattice constants of the material.

The magnetic properties of the particles were determined with a highly sensitive superconducting quantum interference device (SQUID; Quantum Design, MPMS) magnetometer.

## Author Contributions

Z.S.-W. prepared and characterized samples. F.K. and B.R.J. performed the SEM and EDS measurements and contributed to the data interpretation. A.P. helped to analyse the data. M.M. contributed to the discussion. Z.S.-W. and A.P. prepared the manuscript in consultation with all authors.

## Additional Information

**How to cite this article**: Swiatkowska-Warkocka, Z. *et al.* Synthesis of new metastable nanoalloys of immiscible metals with a pulse laser technique. *Sci. Rep.* **5**, 9849; doi: 10.1038/srep09849 (2015).
